# Meta-Analysis of Renal Replacement Therapy for Burn Patients: Incidence Rate, Mortality, and Renal Outcome

**DOI:** 10.3389/fmed.2021.708533

**Published:** 2021-08-09

**Authors:** ZhiYu Duan, GuangYan Cai, JiJun Li, FengKun Chen, XiangMei Chen

**Affiliations:** ^1^State Key Laboratory of Kidney Diseases, Department of Nephrology, National Clinical Research Center for Kidney Diseases, Chinese People's Liberation Army Institute of Nephrology, Chinese People's Liberation Army General Hospital, Beijing, China; ^2^Department of Nephrology, The Fourth Medical Center of People's Liberation Army General Hospital, Beijing, China

**Keywords:** renal replacement therapy, burn patients, acute kidney injury, prevalence, mortality, renal outcome, meta-analysis

## Abstract

**Background:** Renal replacement therapy (RRT) was often needed by some severe burn patients with acute kidney injury (AKI). The primary aim of this study was to review incidence rate and mortality of RRT in severe burn patients. Second aims were to review RRT complications and renal outcome.

**Methods:** We searched multiple databases for studies published between 1 January 1960 and 31 December 2019. Studies about adult populations with burn injury, providing epidemiologic data on prevalence or mortality of RRT, were included.

**Results:** A total of selected 57 studies, including 27,437 patients were enrolled in our analysis. The prevalence rates of RRT were 8.34% (95% CI 7.18–9.5%) in all burn patients and 37.05% (95% CI 29.85–44.24%) in AKI patients. The mortality of all burn patients with RRT was 65.52% (95% CI 58.41–72.64%). The prevalence rates of RRT in sample size≥100 group were 6.86% (95% CI 5.70–8.03%), which was lower than that of <100 group (17.61%, 95% CI 13.39–21.82%). With the increase of TBSA, the prevalence of RRT may have the increasing trend. The prevalence rates of RRT in Asian group was 12.75% (95% CI 9.50–16.00%), which was higher than that of European (10.45%, 95% CI 7.30–13.61%) and North America group (5.61%, 95% CI 4.27–6.95%). The prevalence rates of RRT in 2010–2019 group was 12.22% (95% CI 10.09–14.35%), which was higher than that of 2009–2000 group (5.17%, 95% CI 2.88–7.46%). The prevalence rates of RRT in 1989 and before group was the lowest, which was 1.56% (95% CI 0–3.68%). However, there was no significant correlation between the year of publication and the mortality of burn patients with RRT. Dialysis-requiring AKI in burn patients could increases the risk of chronic kidney disease progression and end-stage renal disease. About 35% of RRT patients need to maintain haemodialysis temporarily, even if they survive and leave hospital.

**Conclusions:** The prevalence rate of RRT is about 6–8%; approximately, one-third of burn patients with AKI need RRT. The prevalence rate of RRT increased over time, but the mortality did not change. The prevalence rates of RRT in Asian group was higher than that of European and North America group.

## Introduction

Acute kidney injury (AKI) is one of the common complications of burn patients that seriously threatens the life of patients and increases the length of stay, intensive care unit (ICU) length of stay and treatment costs (1, 2). In 2010, meta-analysis reported that the prevalence of AKI in burn patients ranged from 16 to 26.6% (3), depending on the severity of the burns and on the definition of AKI. The prevalence of AKI in burn patients ranged from 18.4 to 47.4% with the RIFLE standard (2). Burn patients with AKI had a significantly increased risk of death. The mortality of burn patients with AKI was 16.95–100%, significantly higher than that of their control group (7–29.41%) (4–7).

However, the prevalence and mortality of renal replacement therapy (RRT) for burn patients are still unclear. A meta-analysis in 2010 conducted a subgroup analysis of the prevalence and mortality of RRT in burn AKI patients. The results showed that the prevalence of RRT was 3.2% in all burn patients and 27.1% in burn patients with AKI. The mortality of burn patients with RRT was as high as 80% [95% confidence interval (CI) 72–88.6%] (3). Moreover, the updated meta-analysis of burn patients admitted to the ICU showed that the prevalence of RRT in all burn patients was 12%, and the mortality was 74% (95% CI 58–87%) (8). A recently published multicenter observational study showed that the mortality rate of RRT in severe burn patients has been reduced to about 50% (9). The mortality of burn patients with RRT was still very high. However, due to the different directions of attention, the literature of RRT for burn patients included in these studies is incomplete. At present, there is a lack of meta-analysis of RRT for burn patients. The primary aim of this study was to review incidence rate and mortality of RRT in burn patients. Second aims were to review RRT complications and renal outcome.

## Methods

This systematic review was conducted using the Preferred Reporting Items for Systematic Reviews and Meta-Analyzes and the Meta-Analysis of Observational Studies in Epidemiology guidelines (10, 11).

### Eligibility Criteria

Studies on adult populations with burn injury, providing epidemiologic data on prevalence and mortality of RRT, were included. The adult (>16 years of age) patients with burns covering 10% or more of the total body surface area (TBSA) were enrolled. Moreover, TBSA used in the subgroup analysis. Studies conducted only in patients with chemical or electrical burns were not included because of the different pathophysiologies. We excluded studies with a sample size of <10. Other blood purification techniques, such as plasma exchange (non-RRT), are not included in this study.

### Search Strategy

We used the following terms for standard medical subject headings and free-text words: burn, burns, renal, kidney, kidney injury, kidney diseases, renal insufficiency, renal failure, and kidney failure. We also reviewed the references cited in all the studies selected for review. The search was performed in January 2020 and considered the period 1960–2019. The year of publication used in the subgroup analysis. An extensive search of literature published was conducted using the databases PubMed/MEDLINE, Embase, Science Citation Index (Web of Science), and the Cochrane Central Register of Controlled Trials (CENTRAL) database. Two searchers (D.ZY. and C.FK.) conducted the search independently. If one of the searchers thought that the article was appropriate, the study was selected.

### Study Selection

Two reviews (D.ZY. and C.FK.) independently screened studies for eligibility according to study selection criteria. The inclusion criteria were randomized controlled trial (RCT), case-control and cohort studies. Cross-sectional studies were excluded because they cannot clearly identify the causal relationship between observed indicators and diseases. Only articles in English, Japanese or Chinese were included. Populations with pediatric patients, animal studies, case reports and reviews were excluded. Any disagreement between reviewers were resolved by consensus. RRTs included slow low-efficiency dialysis, intermittent haemodialysis (IHD), peritoneal dialysis (PD), and continuous renal replacement therapy (CRRT). We excluded the study of plasma exchange in burn patients because the mechanism and mode of treatment of plasma exchange are different from those of dialysis. We excluded the lack of mortality studies aimed at studying antibiotic pharmacokinetics in burn patients with RRT. We excluded the pre-existing comorbidity for RRT such as end-stage renal disease. For studies that gave multiple mortalities, we took the longest mortality, such as 28, 60, 90, or in-hospital mortality.

### Study Quality

We assessed risk of bias for RCTs using the Cochrane Collaboration's tool for assessing bias (12). We assessed risk of bias for case-control and cohort studies using the Newcastle-Ottawa Scale (NOS) (13). The NOS allocates nine points for quality of the selection (four items, four points), comparability (one item, two points), and outcome or exposure (three items, three points). Publication bias was assessed by creating and examining funnel plots. The robustness of the results was evaluated using sensitivity analyzes. Each study included in this review was assessed for quality as good (7–9), moderate (4–6), or poor (≤3) based on scores.

### Data Extraction and Statistical Analysis

Two independent reviewers (D.ZY. and C.FK.) extracted the following information from the included studies: title, author, year, journal, study design, nationality, sample size, definition of AKI, number of patients with or without AKI, number of patients with or without RRT, RRT details (modalities, device and manufacturer, dose, anticoagulants), and results (mortality of AKI patients, mortality of RRT patients). We also reported other outcomes (RRT complications, renal outcome-long-term dialysis, temporarily required dialysis, chronic kidney disease progress).

All ambiguities in data extraction were double-checked and resolved. Relative risks and 95% confidence intervals were obtained using a random effects model. *I*^2^ derived from the chi-squared test was used to evaluate the heterogeneity across the included studies. An *I*^2^ <50% indicated that there was no significant heterogeneity (14). Sensitivity analysis was performed by sequentially removing each individual study. We assessed publication bias by constructing a funnel plot. Pearson or Spearman's correlation was used to analyze correlations. A two-tailed *P*-value of <0.05 was considered statistically significant. All statistical analyzes were performed using Review Manager version 5.3, SPSS 24.0 and R 3.5.1 software were used for meta-analyzes of prevalence and mortality.

## Results

### Quality Assessment and Study Characteristics

**Figure 1:**
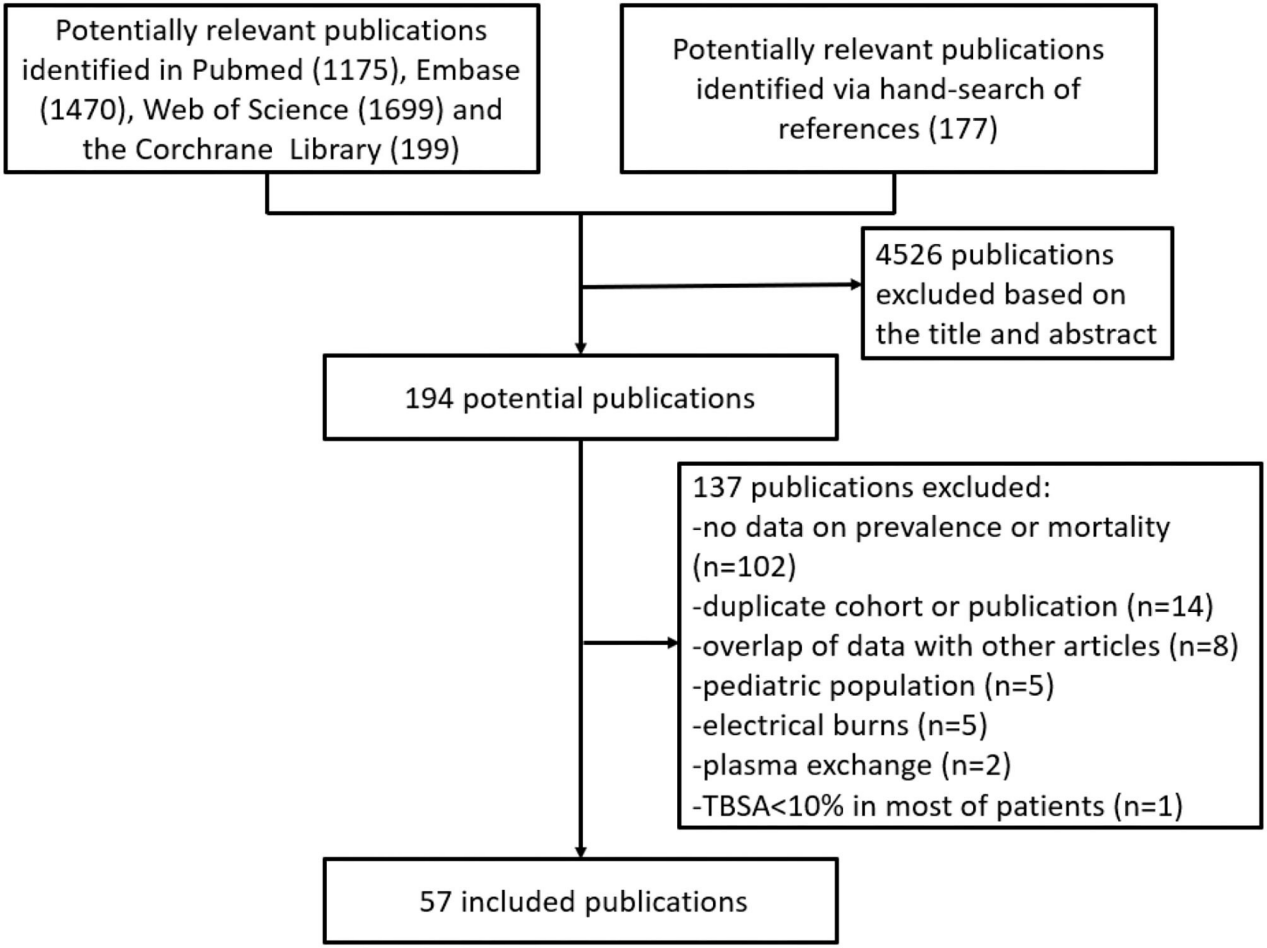
Flow diagram of the study selection process.

We screened and evaluated 4,720 studies, assessing 194 for eligibility. The selected 57 studies, including 27,437 patients, were enrolled in our analysis (Figure 1) (4–7, 9, 15–65). There were 4,345 burn patients with AKI and 1,754 burn patients with RRT. A total of 93.5% of the burn patients with RRT were AKI patients. Figure 1 shows a flow chart of the identification and selection of the studies.

The main features of these studies are shown in Table 1. Risk of bias is summarized in Supplementary Table 1 of the (cohort or case-control studies) and Supplementary Figure 1 of the (RCT studies). Among the 57 included studies, 22 were from North America (4, 9, 15, 16, 24, 26, 28, 30, 31, 33, 34, 40, 42–44, 48–50, 54, 59, 61, 62), 18 were from Asia (5, 7, 19, 21–23, 25, 36–38, 41, 46, 47, 51, 52, 58, 64, 67), 15 were from European countries (6, 17, 18, 20, 27, 32, 35, 45, 53, 55–57, 60, 65, 66), one was from South America (29), one was Africa (39). A total of 12 studies could not be used for analysis of the prevalence of RRT, seven of which only reported on RRT patients (9, 18, 34, 37, 38, 43, 45), four of which were RCT studies (40, 41, 51, 52), and the remaining one of which were historical controls (44). Most of the 40 studies (40/57) were retrospective cohort studies, but 12 were prospective cohort studies (7, 32, 36, 39, 43, 46, 47, 50, 57, 58, 64, 65), and 5 RCT studies (40, 41, 51, 52, 56).

**Table 1:** Summary of the baseline characteristics of the studies included in the meta-analysis.

**Study**	**Study type**	**Sample size**	**AKI definition**	**AKI numbers**	**RRT numbers**	**RRT mortality (%)**
Akers et al. (42)	Retrospective cohort	171	≥0.5 mg/dL SCr increase	38	33	19 (57.58)
Bechir et al. (57)	Prospective cohort	30	Dialysis	5	5	NA
Bechir et al. (56)	Randomized controlled trial	45	Dialysis	12	12	NA
Boucher et al. (43)	Prospective cohort	10	AKIN criteria	9	10	3 (30)
Chrysopoulo et al. (15)	Retrospective cohort	1,404	Oliguria for at least 36 h (urine output <350 mL/d), a blood urea nitrogen-creatinine ratio of <20, Scr>2 mg/dL, and the requirement for dialysis after injury	76	67	61 (91.04)
Chun et al. (58)	Prospective cohort	76	AKIN criteria	32	20	19 (95)
Chung et al. (44)	Retrospective cohort	102	RIFLE classification	34	18	10 (55.56)
Chung et al. (40)	Randomized controlled trial	37	Oliguria (<20 ml/h) for >24 h or an increase Scr>2 mg/dl in males or >1.5 mg/dl in females over a period of <4 days	37	37	23 (62.16)
Chung et al. (9)	Retrospective cohort	4,086	KDIGO criteria	160	170	85 (50)
Clark et al. (59)	Retrospective cohort	639	KDIGO criteria	422	49	33 (67.35)
Coca et al. (4)	Retrospective cohort	304	RIFLE classification	81	11	8 (72.73)
Damkat-Thomas et al. (16)	Retrospective cohort	41	RIFLE classification	17	5	2 (40)
Davies et al. (17)	Retrospective cohort	1,064	NA	28	25	22 (88)
Demsey et al. (54)	Retrospective cohort	151	AKIN criteria	64	18	7 (38.89)
Depret et al. (60)	Retrospective cohort	87	KDIGO criteria	55	21	NA
Gille et al. (18)	Retrospective cohort	18	NA	18	18	2 (11.11)
Haberal et al. (19)	Retrospective cohort	915	NA	19	19	15 (78.95)
Hladik et al. (45)	Retrospective cohort	40	NA	10	40	28 (70)
Holm et al. (20)	Retrospective cohort	328	Scr >2.0 mg/dl (with rising tendency) combined with a blood urea nitrogen level >200 mg/dl or in patients with anuria or oliguria (urine volume <400 ml/24 h)	48	48	41 (85.42)
Hong et al. (46)	Prospective cohort	45	RIFLE classification	11	5	4 (80)
Hu et al. (21)	Retrospective cohort	396	RIFLE classification	151	25	NA
Hundeshagen et al. (61)	Retrospective cohort	246(adults)	KDIGO criteria	26	3	NA
Kim et al. (5)	Retrospective cohort	147	Scr ≥2 mg/dL	28	3	3 (100)
Kumar et al. (62)	Retrospective cohort	254	AKIN criteria	190	10	NA
Kuo et al. (22)	Retrospective cohort	145	KDIGO criteria	59	9	7 (77.78)
Kuo et al. (23)	Retrospective cohort	301	AKIN criteria	34	28	NA
Kym et al. (47)	Prospective cohort	85	RIFLE classification	48	22	NA
Leblanc et al. (24)	Retrospective cohort	970	NA	16	16	13 (81.25)
Liu et al. (52)	Randomized controlled trial	41	NA	NA	20	7 (35)
Liu (25)	Retrospective cohort	6,050	NA	53	15	8 (53.33)
Lopes et al. (53)	Retrospective cohort	126	Doubling of baseline Scr	32	11	NA
Mariano et al. (55)	Retrospective cohort	548	NA	98	70	50 (71.43)
Mason et al. (26)	Retrospective cohort	330	Scr >1.5 mg/dL	48	37	NA
Munoz et al. (6)	Retrospective cohort	840	KDIGO criteria	466	34	NA
Mustonen and Vuola (27)	Retrospective cohort	238	Scr >120 umol/L (1.4 mg/dL); for chronic renal insufficiency patients, 2-fold rise in Scr or Scr rose >100 μmol/l during 1 day	93	32	20 (62.5)
Peng et al. (51)	Randomized controlled trial	20	NA	NA	10	1 (10)
Planas et al. (48)	Retrospective cohort	29	Scr level above initial values to a level equal to or >1.5 mg/dL	11	3	2 (66.67)
Pronina et al. (28)	Retrospective cohort	1,405	AKIN or RIFLE criteria	53	21	7 (33.33)
Queiroz et al. (29)	Retrospective cohort	293	An elevation in baseline serum creatinine greater than or equal to 50% from baseline	77	52	NA
Rakkolainen et al. (66)	Retrospective cohort	187	Scr ≥120 umol/L (1.4 mg/dl)	51	21	9 (42.86)
Ren et al. (64)	Prospective cohort	58	KDIGO criteria	11	5	4 (80)
Sabry et al. (39)	Prospective cohort	40	Scr>2 mg/dL and blood urea nitrogen>25 mg/dL	9	4	2 (50)
Saffle et al. (30)	Retrospective cohort	529	Scr>132.6 umol/L (1.5 mg/dL)	143	5	5 (100)
Sanchez-Sanchez et al. (65)	Prospective cohort	165	RIFLE classification	32	15	14 (93.33)
Schneider et al. (49)	Retrospective cohort	220	RIFLE classification	103	25	NA
Sen et al. (50)	Prospective cohort	30	RIFLE classification	14	3	NA
Soltani et al. (31)	Retrospective cohort	1,125	NA	38	33	23 (69.7)
Steinvall et al. (32)	Prospective cohort	127	RIFLE classification	31	4	3 (75)
Stewart et al. (33)	Retrospective cohort	1,967	AKIN criteria	640	70	49 (70)
Tang et al. (67)	Retrospective cohort	157	AKIN criteria	89	82	NA
Tremblay et al. (34)	Retrospective cohort	12	NA	12	12	6 (50)
Witkowski etal. (35)	Retrospective cohort	225	Decrease in GFR of <60 ml/min at admission, decrease in GFR of more than 75% compared to baseline or decrease in the daily diuresis of <500 ml for at least 24 h	135	9	9 (100)
Yang et al. (36)	Prospective cohort	90	RIFLE classification	55	22	17 (77.27)
Yim et al. (7)	Prospective cohort	97	AKIN criteria	40	23	NA
Yoon et al. (37)	Retrospective cohort	84	RIFLE classification	84	84	71 (84.5)
Yoon et al. (38)	Retrospective cohort	216	AKIN criteria	190	216	176 (81.48)
You (41)	Randomized controlled trial	82	KDIGO stage 3	9	41	11 (26.83)

*AKI, acute kidney injury; RRT, renal replacement therapy; NA, not available; Scr, serum creatine; GFR, glomerular filtration rate*.

### Prevalence and Mortality of RRT in Burn Patients

We analyzed 45 literatures that reported the prevalence of RRT in burn patients (Table 2) (4–7, 15, 16, 19–33, 35, 36, 39, 42, 46–50, 53–62, 64–68). The prevalence rates of RRT were 8.34% (95% CI 7.18–9.5%) in all burn patients and 37.05% (95% CI 29.85–44.24%) in AKI patients. The prevalence of RRT among burn patients in the ICU was 11.14% (95% CI 8.86–13.42%) (4–7, 16, 22, 27, 29, 31, 36, 42, 46, 47, 50, 54, 56, 57, 59, 60, 62, 65, 66). A total of 25 studies with RIFLE, AKIN and KDIGO as AKI diagnostic criteria after 2004 were analyzed (4, 6, 7, 16, 21–23, 28, 32, 33, 35, 36, 46, 47, 49, 50, 54, 58–62, 64, 65, 67). The prevalence of RRT in these burn patients was 30.03% (95% CI 23.88–36.18%).

**Table 2:** The prevalence of RRT in burn patients with different diagnostic criteria.

**Diagnosis**	**N. of Trials**	**Patients**	***I*^**2**^ (%)**	***P***	**Prevalence (%)**	**95% CI**
All burn patients	45	22,726	96	<0.01	8.34	7.18–9.5
RRT of AKI patients	38	3,556	98	<0.01	37.05	29.85–44.24
ICU	22	5,480	90	<0.01	11.14	8.86–13.42
RIFLE classification	11	596	78	<0.01	28.85	21.0–36.69
AKIN classification	9	1,180	98	<0.01	43.96	28.0–59.91
KDIGO classification	6	1,039	84	<0.01	15.97	9.51–22.44
Summary of RIFLE, AKIN, KDIGO	25	2,859	95	<0.01	30.03	23.88–36.18

*CI, confidence interval; RRT, renal replacement therapy; AKI, acute kidney disease; ICU, intensive care unit*.

In order to balance the bias of small sample study (*n* < 100), we did subgroup analysis for the study with sample size >100 (Supplementary Table 2). The prevalence rates of RRT in these studies were 6.86% (95% CI 5.70–8.03%), which was lower than that of sample size <100 group (17.61%, 95% CI 13.39–21.82%). The prevalence rates of RRT in sample size ≥1,000 group were 2.52% (95% CI 1.02–4.02%). However, one third of the studies in sample size ≥1,000 group were from the 1980s, and the prevalence rates of RRT may be reduced because RRT was still in its infancy at that time.

The TBSA was used for subgroup analysis (≥10%, ≥20%, ≥30% or second and third degree burns >10%, ≥40% or second and third degree burns >20%). The results showed that, with the increase of TBSA, the prevalence of RRT may have the increasing trend (Supplementary Table 3). The prevalence rates of RRT in TBSA ≥20% group was 14.33% (95% CI 10.10–18.57%), which was higher than that of TBSA ≥10% group (6.4%, 95% CI 4.12–8.69%). The prevalence rates of RRT in TBSA ≥40% group was the highest, which was 14.66% (95% CI 5.22–24.09%).

In the study location subgroup analysis (Supplementary Table 4), the prevalence rates of RRT in Asian group was 12.75% (95% CI 9.50–16.00%), which was higher than that of European (10.45%, 95% CI 7.30–13.61%) and North America group (5.61%, 95% CI 4.27–6.95%).

Moreover, we analyzed the results of 41 studies that reported RRT mortality in burn patients (Table 3). The mortality of all burn patients with RRT was 65.52% (95% CI 58.41–72.64%) (4, 5, 9, 15–20, 22, 24, 25, 27, 28, 30–46, 48, 51, 52, 54, 55, 58, 59, 64–66). The mortality of patients with RRT in ICU was 62.7% (95% CI 53.7–71.7%) (4, 5, 9, 16, 22, 27, 31, 36, 38, 40–46, 54, 59, 65, 66). The results of 20 studies with RIFLE, AKIN, and KDIGO as AKI diagnostic criteria showed that the mortality of RRT in burn patients was 67.16% (95% CI 57.40–76.93%) (4, 9, 16, 22, 28, 32, 33, 35–38, 41, 43, 44, 46, 54, 58, 59, 64, 65). Three studies reported deaths in all burn patients undergoing RRT (5, 30, 35, 42). According to different mortality categories, the mortalities of 14 days, 28 days and 60 days ranged from 30 to 50%, while those of ICU and hospital were 56.98 and 68.89%, and overall mortality further increased to 75.24% (Supplementary Table 5).

**Table 3:** The mortality of RRT in burn patients with different diagnostic criteria.

**Diagnosis**	**N. of Trials**	**Patients**	***I*^**2**^ (%)**	***P***	**RRT mortality (%)**	**95% CI**
Summary of all literatures	41	1,342	90	<0.01	65.52	58.41–72.64
ICU	20	797	85	<0.01	62.7	53.7–71.7
RIFLE classification	9	185	77	<0.01	70.08	56.4–83.75
AKIN classification	7	370	90	<0.01	66.73	52.01–81.45
KDIGO classification	5	283	81	<0.01	55.29	39.46–71.12
Summary of RIFLE, AKIN, KDIGO	20	811	90	<0.01	67.16	57.40–76.93

*RRT, renal replacement therapy; CI, confidence interval; ICU, intensive care unit*.

According to the three diagnostic criteria of RIFLE, AKIN, and KDIGO, the prevalence of RRT was KDIGO < RIFLE < AKIN, and that of mortality was KDIGO < AKIN < RIFLE. The prevalence of RRT was 15.97% (95% CI 9.51–22.44%) and that of mortality was 55.29% (95% CI 39.46–71.12%) in the six literatures with KDIGO classification as the diagnostic standard, which was lower than other AKI diagnostic standards.

There was no significant correlation (*r* = −0.224, *P* = 0.159) between the year of publication and the mortality of burn patients with RRT (Supplementary Figure 2). According to the year of publication, the patients were divided into four subgroups (Supplementary Figure 3) from 2010 to 2020, 2000 to 2009, 1990 to 1999, 1989 and before. The mortality of the 2010–2020 group was 60.42 ± 25.35%, that of the 2000–2009 group was 61.55 ± 23.29%, that of the 1990–1999 group was 87.33 ± 8.44%, and that of the 1989 and before group was 63.52 ± 25.05%. There was no significant difference between groups (*P* = 0.139). After 2010, three studies still reported that the mortality of RRT patients was more than 90% (35, 58, 65). The prevalence rates of RRT in 2010–2019 group was 12.22% (95% CI 10.09–14.35%), which was higher than that of 2009–2000 group (5.17%, 95% CI 2.88–7.46%). The prevalence rates of RRT in 1989 and before group was the lowest, which was 1.56% (95% CI 0–3.68%). The results showed that the prevalence rate of RRT increased over time (Supplementary Table 6).

### RRT-Related Adverse Reactions

Nine articles reported the incidence of RRT-related adverse reactions. The total incidence was 28.77% (63/229) (18, 20, 24, 25, 27, 34, 40, 41, 51), including thrombocytopenia 0.44% (1/229), bleeding 10.92% (25/229), thromboembolism 1.75% (4/229), secondary infection 9.61% (22/229), electrolyte disorder 2.62% (6/229), and imbalance syndrome 0.44% (1/229). Among them, only Chung 2017 reported six patients with electrolyte disorder. Other literatures may not mention the occurrence of electrolyte disorder due to certain concerns, which may underestimate the prevalence of electrolyte disorder. A total of 16 patients with PD were reported in 9 articles; most of these patients were from Liu 1986 (25). Among the 16 patients, 1 had “unbalanced syndrome” and improved after stopping dialysis; 4 had abdominal infection, 3 survived, and one changed to haemodialysis and ultimately died. Considering the immature technology at that time, the incidence of PD adverse reactions may be overestimated.

### Renal Outcome

A recent study found an odds of dialysis of 2.40 in burn patients who developed AKI compared with the general Finnish population (69). Eleven studies followed up the long-term renal outcomes of burn patients who survived RRT (9, 16, 18, 24, 27, 31, 33, 34, 40, 50, 54). A total of 184 patients survived after RRT; 64.13% of them (118/184) were dialysis-independent after discharge, 25% (46/184) needed temporary required dialysis, and 10.87% (20/184) needed long-term dialysis (more than 6 months after discharge). Thalji 2017 found that 1 year after burn, the proportion of chronic dialysis in non-AKI patients was 0.33% (56/16985), significantly lower than that in patients with AKI, which was 4.58% (26/568) (70). The proportion of severe chronic kidney disease (CKD, defined as stage 3–5) in non-AKI patients (0.71%) was also lower than those in AKI patients (5.81%) (70). Gille reported that 3 of the 16 surviving burn patients undergoing CRRT had CKD progression (glomerular filtration rate (GFR) < 45 ml/min.1.73 m^2^, CKD 3b) (18.75%) (18). Two patients had slightly impaired GFR (< 90 ml/min.1.73 m^2^, CKD 2) before the burn trauma. One patient had normal GFR (18).

## Discussion

Acute kidney injury is one of the common complications in burn patients. The incidence of AKI varied from 16% to 26.6% according to the definition of AKI in different populations (3). The incidence of AKI in burn ICUs was 38% (30–46%) (8). RRT was often needed by some severe burn patients with AKI. According to the subgroup analysis in the previous meta-analysis of burn patients with AKI, the proportion of all burn patients requiring RRT was ~3% (3). The proportion of burn patients admitted to the ICU was 12% (8–16%) (8), and the mortality was 74% (95% CI 58–87%) (3). A recently published multicenter observational study showed that the mortality rate of RRT in severe burn patients has been reduced to about 50% (9). The mortality of burn patients with RRT was still very high. Such high mortality rates were the reasons we wanted to conduct a meta-analysis of RRT in burn patients. A total of 27,437 burn patients were enrolled in this study from 57 literatures from 1979 to 2019. The results showed that the prevalence rates of RRT were 8.34% (95% CI 7.18–9.5%) in all burn patients and 37.05% (95% CI 29.85–44.24%) in AKI patients. These data are higher than those reported in the subgroup of previous studies (3, 8). Due to the different purposes of previous studies, the number of RRT studies included in previous studies was significantly lower than that of this study.

The study of small sample size may enlarge the observation effect, which was first described by Sterne et al. (71). Small trials are more likely to report larger beneficial effects than large trials in critical care medicine (72). This study also found that compared with the large sample size study (*n* ≥ 100), the small sample size study may significantly enlarge the observation effect, that is, the prevalence rates of RRT. However, since the prevalence rates of RRT is not high in all burn patients, and some small sample size studies can provide more information on mortality, adverse reactions and renal outcome in RRT patients, we did not exclude small sample study, but instead, we used subgroup analysis to list them separately.

With the increase of TBSA, the risk of AKI is greater, and the risk of RRT may also be greater. High TBSA was a risk factor for AKI in burn patients (8). Our results showed that, with the increase of TBSA, the prevalence rates of RRT may have the increasing trend. The prevalence rates of RRT in TBSA ≥40% group was the highest. In a retrospective study of the aluminum dust explosion accident in Kunshan factory, 157 patients with severe burns were included, with an average TBSA more than 90%. In this study, the incidence of AKI was 56.7%, while the prevalence rates of RRT was 52.2% (67). In the study location subgroup analysis, the prevalence rates of RRT in Asian group was 12.75%, which was higher than that of European and North America group. The reason for the increased the prevalence rates of RRT in Asian group may be closely related to the large number of small samples studies (about 50%, 6/13) and the large TBSA of patients (TBSA ≥40% goup, 2/5).

With the improvement and popularization of RRT, more and more severe burn patients with AKI can be treated with RRT. Our results showed that with the progress of the times, the prevalence rates of RRT in burn patients is gradually increasing from <2% before 1980s to 12.22% now. Before CRRT was popularized, some severe burn patients with AKI were unable to tolerate IHD because of hemodynamic compromise. Chung et al. reported in 2008 that their center started CRRT in November 2005. Compared with the CRRT group, none of the 16 patients in the consecutive historical control group matched with TBSA and injury severity score were placed on haemodialysis (44). In Chinese mainland, only 15 of 6,050 burn patients underwent RRT in the 1980s, and most of them were PD. About 1/4 of the patients receiving PD developed abdominal infection (25). In 2012, the number rose to 6.31%, and all of them received CRRT treatment (21). Moreover, economic reasons may also affect whether burn patients start RRT. A survey of nephrologists and critical care physicians in 189 different hospitals in China showed that one of the reasons prohibiting patients from getting RRT were high therapy costs (73). With the development of economy and the improvement of medical insurance reimbursement policy in recent years, the prevalence rate of RRT in severe burn patients with AKI may also increase. CRRT could make more severe burn patients with an indication for RRT and hemodynamic instability have the opportunity received RRT treatment. However, RCT and meta-analysis studies in critically ill patients did not find that CRRT had a survival benefit advantage compared with IHD and PD in hemodynamic stable patients (74, 75). There was no significant correlation between the year of publication and the mortality of burn patients with RRT in this study. Patients in the 1980 and 1990 groups received peritoneal dialysis or haemodialysis. Compared with the 1989 and before group and 1990–1999 group (received IHD or PD), most patients in the 2010–2020 group received CRRT, but there was no significant difference in mortality between groups.

RIFLE (76), AKIN (77) and KDIGO (78) are three commonly used AKI grading standards after 2004. The KDIGO diagnostic standard proposed in 2012 had been reported to improve the early diagnosis of kidney injury (59, 79) and to reduce the missed diagnosis rate (80), which was conducive to the early diagnosis and treatment of AKI. Our results showed that, compared with RIFLE and AKIN, the prevalence and mortality of RRT in KDIGO were relatively low. We speculate that early diagnosis and early treatment may help some burn patients with AKI avoid further deterioration of renal function, reduce the need for dialysis, and improve the prognosis of patients. Ren et al., defined AKI according to KDIGO guidelines by 48 h after admission. That prospective study reported 11.2% (11/95) AKI and only 35.4% mortality in the AKI group (64).

AKI in burn patients not only increases mortality (81), but also increases the risk of CKD progression and end-stage renal disease (69, 70). When severe burn patients have dialysis-requiring AKI, ~35% of the patients need to maintain haemodialysis temporarily, even if they survive and leave the hospital. Among them, 10% of the surviving patients need haemodialysis for more than half a year. Therefore, for burn AKI or dialysis-requiring AKI survivors, it is recommended to monitor the renal function regularly within 1 year after discharge and to avoid the use of nephrotoxic drugs, excessive diuresis, and diarrhea (82), to reduce the progress of CKD and to avoid the recurrence of AKI.

This systematic review has several limitations. First, there was significant heterogeneity in most results of RRT prevalence and mortality in this study. The clinical heterogeneity of this study may be due to the differences in the study population, RRT modalities and the end-point indicators observed in the study. The inclusion and exclusion criteria were different among different studies. There were controversies on modalities, timing, and dosage of RRT, and the standards reported in different literatures are not the same. Outcome variables, such as mortality, also varied from study to study. Although we conducted a series of subgroup analyzes on AKI diagnostic criteria, ICU, different mortality criteria, TBSA, sample size, study location, and publication year, the results still showed heterogeneity. Because we were unable to obtain the original data for each patient, we cannot completely control the above confounding factors. Therefore, we used the random effect model to analyze the results. Second, we only included documents in English, Japanese and Chinese, and there may be omissions in documents reported in other languages, such as Russian. There was no literature from Russia in the studies we included. However, 57 articles and nearly 30,000 burn patients from five continents were included in this meta-analysis, most of which were in North America, Asia, and Europe. The inclusion of a large number of literatures and patients can ensure that the results of this study were well-representative, especially in North America, Asia, and Europe. Third, this study did not analyze the starting time of RRT for burn patients with AKI. At present, the timing of RRT for critically ill patients is still controversial. At the beginning of this study, we plan to analyze the timing of RRT in burn patients. However, even if 57 articles were included, there is still a lack of head-to-head study on the starting time of RRT for burn patients with AKI. There was only one cohort study that was divided into groups according to the AKI level of patients at the beginning of CRRT. The early CRRT group was defined according to the patients who started CRRT at the risk stage of the RIFLE criteria. The results showed there were no significant differences in mortality by the severity of AKI at the time of CRRT initiation (37). We suggest that more head-to-head cohort studies or RCT studies can be designed in the future regarding the timing of RRT in burn patients. Finally, the current study is not registered, and there may be a small deviation, but we still strictly follow the steps of systematic review.

Our meta-analysis had a number of strengths. In this study, 57 articles from 1979 to 2019 were included, involving nearly 30,000 burn patients. It has obvious representative advantages for the prevalence and mortality of RRT for burn patients with high statistical variability. Due to the large number of included literatures, we were able to conduct a series of subgroup analyzes on AKI diagnostic criteria, ICU, different mortality criteria, TBSA, sample size, study location, and publication year. As a result of a series of subgroup analysis, we found that The prevalence rate of RRT increased with TBSA and time. The prevalence rates of RRT in Asian group was higher than that of European and North America group.

## Conclusions

The prevalence rate of RRT is about 6–8%; approximately, one-third of burn patients with AKI need RRT. The prevalence rate of RRT increased over time, but the mortality did not change. The prevalence rates of RRT in Asian group was higher than that of European and North America group.

## Data Availability Statement

The original contributions presented in the study are included in the article/Supplementary Material, further inquiries can be directed to the corresponding authors.

## Author Contributions

GC and JL contributed to the conception and design of the study. ZD and FC selected the studies, extracted the data, performed the statistical analyzes, and data presentation. ZD evaluated the study quality. The manuscript was drafted by ZD, with assistance by FC and JL, and the manuscript was critically revised by XC and GC. All authors contributed to the article and approved the submitted version.

## Conflict of Interest

The authors declare that the research was conducted in the absence of any commercial or financial relationships that could be construed as a potential conflict of interest.

## Publisher's Note

All claims expressed in this article are solely those of the authors and do not necessarily represent those of their affiliated organizations, or those of the publisher, the editors and the reviewers. Any product that may be evaluated in this article, or claim that may be made by its manufacturer, is not guaranteed or endorsed by the publisher.
