# Supplementary material for: Meta-Analysis of Renal Replacement Therapy for Burn Patients: Incidence Rate, Mortality, and Renal Outcome
Source: Front Med (Lausanne). 2021 Aug 9;8:708533. doi: 10.3389/fmed.2021.708533 (PMC8381047; doi:10.3389/fmed.2021.708533)
Supplement: Supplementary file 1 [file Data_Sheet_1.docx]

**Supplementary Table 1.** Quality assessment of cohort studies.

|  | Representativeness | Selection of the Non-Exposed | Ascertainment of Exposure | Demonstration That Outcome of Interest Was Not Present at Start of Study | Comparability | Assessment of Outcome | Was Follow-Up Long Enough for Outcomes to Occur | Adequacy of Follow Up of Cohorts | Total scores |
| --- | --- | --- | --- | --- | --- | --- | --- | --- | --- |
| **Retrospective cohort** |  |  |  |  |  |  |  |  |  |
| Akers 2012 | Yes | No | Yes | Yes | No | Yes | Yes | Yes | 6 |
| Chrysopoulo 1999 | Yes | No | Yes | Yes | No | Yes | Yes | No | 5 |
| Chung 2008 | Yes | Yes | Yes | Yes | Yes | Yes | Yes | Yes | 8 |
| Chung 2018 | Yes | No | Yes | Yes | No | Yes | Yes | Yes | 6 |
| Clark 2019 | Yes | Yes | Yes | Yes | Yes | Yes | Yes | Yes | 8 |
| Coca 2007 | Yes | Yes | Yes | Yes | No | Yes | Yes | Yes | 7 |
| Damkat-Thomas 2011 | Yes | Yes | Yes | Yes | No | Yes | Yes | No | 6 |
| Davies 1979 | Yes | No | Yes | Yes | No | Yes | Yes | Yes | 6 |
| Demsey 2019 | Yes | Yes | Yes | Yes | Yes | Yes | Yes | Yes | 8 |
| Dépret 2018 | Yes | Yes | Yes | Yes | No | Yes | Yes | Yes | 7 |
| Gille 2014 | Yes | No | Yes | Yes | No | Yes | Yes | Yes | 6 |
| Haberal 1993 | Yes | No | Yes | Yes | No | Yes | Yes | Yes | 6 |
| Hladik 2001 | Yes | No | Yes | Yes | No | Yes | Yes | Yes | 6 |
| Holm 1999 | Yes | No | Yes | Yes | No | Yes | Yes | Yes | 6 |
| Hu 2012 | Yes | Yes | Yes | Yes | No | Yes | Yes | Yes | 7 |
| Hundeshagen 2017 | Yes | Yes | Yes | Yes | No | Yes | Yes | Yes | 7 |
| Kim 2003 | Yes | Yes | Yes | Yes | Yes | Yes | Yes | Yes | 8 |
| Kumar 2016 | Yes | Yes | Yes | Yes | No | Yes | Yes | Yes | 7 |
| Kuo 2016 | Yes | Yes | Yes | Yes | No | Yes | Yes | Yes | 7 |
| Kuo 2018 | Yes | Yes | Yes | Yes | No | Yes | Yes | Yes | 7 |
| Leblanc 1997 | Yes | Yes | Yes | Yes | Yes | Yes | Yes | Yes | 8 |
| Liu 1986 | Yes | No | Yes | Yes | No | Yes | Yes | Yes | 6 |
| Lopes 2007 | Yes | Yes | Yes | Yes | No | Yes | Yes | No | 6 |
| Mariano 2010 | Yes | Yes | Yes | Yes | No | Yes | Yes | Yes | 7 |
| Mason 2016 | Yes | Yes | Yes | Yes | Yes | Yes | Yes | Yes | 8 |
| Muñoz 2017 | Yes | Yes | Yes | Yes | Yes | Yes | Yes | Yes | 8 |
| Mustonen 2008 | Yes | Yes | Yes | Yes | Yes | Yes | Yes | Yes | 8 |
| Planas 1982 | Yes | Yes | Yes | Yes | No | Yes | Yes | Yes | 7 |
| Pronina 2015 | Yes | Yes | Yes | Yes | Yes | Yes | Yes | Yes | 8 |
| Queiroz 2016 | Yes | No | Yes | Yes | No | Yes | Yes | Yes | 6 |
| Rakkolainen 2018 | Yes | Yes | Yes | Yes | Yes | Yes | Yes | Yes | 8 |
| Saffle 1993 | Yes | No | Yes | Yes | No | Yes | Yes | Yes | 6 |
| Schneider 2012 | Yes | Yes | Yes | Yes | No | Yes | Yes | Yes | 7 |
| Soltani 2009 | Yes | No | Yes | Yes | No | Yes | Yes | Yes | 6 |
| Stewart 2013 | Yes | Yes | Yes | Yes | Yes | Yes | Yes | Yes | 8 |
| Tang 2018 | Yes | No | Yes | Yes | Yes | Yes | Yes | Yes | 7 |
| Tremblay 2000 | Yes | No | Yes | Yes | No | Yes | Yes | Yes | 6 |
| Witkowski 2016 | Yes | Yes | Yes | Yes | No | Yes | Yes | Yes | 7 |
| Yoon 2017-Burns | Yes | No | Yes | Yes | No | Yes | Yes | Yes | 6 |
| Yoon 2017-PLOS ONE | Yes | Yes | Yes | Yes | Yes | Yes | Yes | Yes | 8 |
| **Prospective cohort** |  |  |  |  |  |  |  |  |  |
| Béchir 2010 | Yes | Yes | Yes | Yes | No | Yes | Yes | Yes | 7 |
| Boucher 2016 | Yes | Yes | Yes | Yes | No | Yes | Yes | Yes | 7 |
| Chun 2018 | Yes | Yes | Yes | Yes | Yes | Yes | Yes | Yes | 8 |
| Hong 2013 | Yes | Yes | Yes | Yes | No | Yes | Yes | No | 6 |
| Kym 2015 | Yes | Yes | Yes | Yes | No | Yes | Yes | No | 6 |
| Ren 2015 | Yes | Yes | Yes | Yes | Yes | Yes | Yes | Yes | 8 |
| Sabry 2009 | Yes | Yes | Yes | Yes | No | Yes | Yes | Yes | 7 |
| Sánchez-Sánchez 2016 | Yes | Yes | Yes | Yes | Yes | Yes | Yes | Yes | 8 |
| Sen 2015 | Yes | Yes | Yes | Yes | Yes | Yes | Yes | Yes | 8 |
| Steinvall 2008 | Yes | Yes | Yes | Yes | No | Yes | Yes | No | 6 |
| Yang 2014 | Yes | Yes | Yes | Yes | No | Yes | Yes | Yes | 7 |
| Yim 2015 | Yes | Yes | Yes | Yes | No | Yes | Yes | No | 6 |

**Supplementary Table 2.** The prevalence of RRT in burn patients with different sample size.

| Sample size | N. of Trials | Patients | I^2^ (%) | P | Prevalence (%) | 95%CI |
| --- | --- | --- | --- | --- | --- | --- |
| <100 | 13 | 753 | 59 | < 0.01 | 17.61 | 13.39-21.82 |
| ≥100 | 32 | 21973 | 97 | < 0.01 | 6.86 | 5.70-8.03 |
| >1000 | 6 | 13021 | 97 | < 0.01 | 2.52 | 1.02-4.02 |

CI, confidence interval; RRT, renal replacement therapy.

**Supplementary Table 3.** The prevalence of RRT in burn patients with different total body surface area.

| TBSA | N. of Trials | Patients | I^2^ (%) | P | Prevalence (%) | 95%CI |
| --- | --- | --- | --- | --- | --- | --- |
| ≥10% | 7 | 3269 | 86 | < 0.01 | 6.40 | 4.12-8.69 |
| ≥20% | 13 | 2109 | 89 | < 0.01 | 14.33 | 10.10-18.57 |
| ≥30% or second and third degree burns>10% | 5 | 2498 | 84 | < 0.01 | 6.86 | 4.09-9.63 |
| ≥40% or second and third degree burns＞20% | 5 | 1343 | 98 | < 0.01 | 14.66 | 5.22-24.09 |

TBSA, total body surface area; CI, confidence interval.

**Supplementary Table 4.** The prevalence of RRT in burn patients with different study locations.

| Location | N. of Trials | Patients | I^2^ (%) | P | Prevalence (%) | 95%CI |
| --- | --- | --- | --- | --- | --- | --- |
| Asian | 13 | 8562 | 97 | < 0.01 | 12.75 | 9.50-16.00 |
| European | 13 | 4010 | 93 | < 0.01 | 10.45 | 7.30-13.61 |
| North America | 17 | 9457 | 90 | < 0.01 | 5.61 | 4.27-6.95 |

CI, confidence interval.

**Supplementary Table 5.** Mortality classification of burn patients.

| Classification | N. of Trials | Patients | I^2^ (%) | P | RRT mortality (%) | 95%CI |
| --- | --- | --- | --- | --- | --- | --- |
| 14-day mortality | 2 | 52 | 82 | 0.02 | 34.32 | 0.78-67.86 |
| 28-day mortality | 5 | 121 | 97 | < 0.01 | 48.66 | 11.65-85.66 |
| 60-day mortality | 2 | 61 | 0 | 0.52 | 29.25 | 17.87-40.63 |
| ICU mortality | 2 | 102 | 0 | 0.38 | 68.89 | 59.95-77.84 |
| In-hospital mortality | 16 | 451 | 79 | < 0.01 | 56.98 | 46.59-67.36 |
| Overall mortality | 18 | 688 | 85 | < 0.01 | 75.24 | 67.13-83.35 |

RRT, renal replacement therapy; CI, confidence interval; RRT, renal replacement therapy.

**Supplementary Table 6.** The prevalence of RRT in burn patients with different publication year.

| Publication year | N. of Trials | Patients | I^2^ (%) | P | Prevalence (%) | 95%CI |
| --- | --- | --- | --- | --- | --- | --- |
| 1989 and before | 3 | 7143 | 91 | < 0.01 | 1.56 | 0-3.68 |
| 1999-1990 | 5 | 4146 | 94 | < 0.01 | 3.89 | 1.87-5.92 |
| 2009-2000 | 7 | 2107 | 80 | < 0.01 | 5.17 | 2.88-7.46 |
| 2020-2010 | 30 | 9330 | 95 | < 0.01 | 12.22 | 10.09-14.35 |

CI, confidence interval.

**Supplementary Figure 1.** Risk of bias of randomized controlled trial.


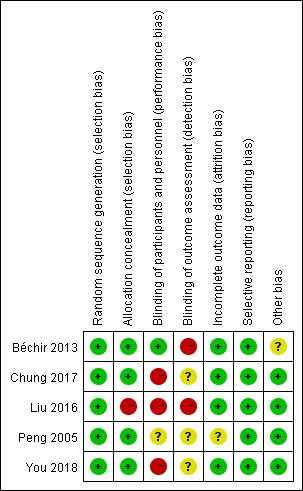


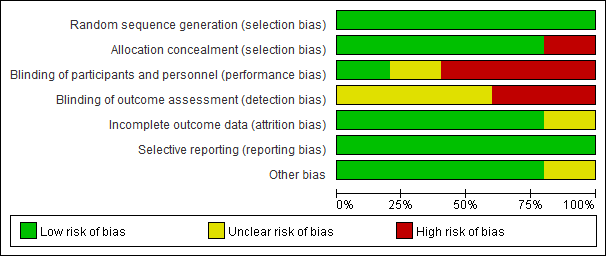


**Supplementary Figure 2.** The correlation analysis between the mortality of renal replacement therapy patients and the publication year.


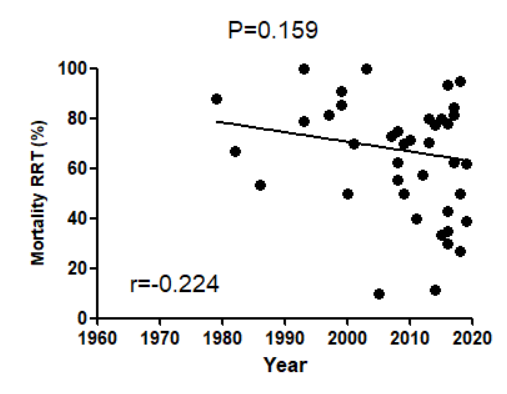


**Supplementary Figure 3.**  Comparison of the mortality of renal replacement therapy patients among subgroups in different years.
